# Ultrasonographic Evaluation of Acute Scrotal Emergencies: A Comprehensive Case Series

**DOI:** 10.7759/cureus.98154

**Published:** 2025-11-30

**Authors:** Christopher D Louviere, Lourdhu P Onteddu, Ruben Ortiz, Oswaldo A Guevara Tirado, Sai R Vulasala, Jennifer L Wen, Neal Hall, Renato Abu Hana, Grit Adler, Dheeraj R Gopireddy

**Affiliations:** 1 Radiology, University of Florida College of Medicine – Jacksonville, Jacksonville, USA

**Keywords:** emergency, scrotal edema, scrotum, testicular, testicular injury, ultrasound imaging

## Abstract

Acute scrotal pain is commonly reported by male patients in emergency settings and comprises a wide range of potential pathological causes. An accurate and expedient diagnosis is essential to avoid treatment and management delays. As such, recognizing imaging features of these clinical conditions is vital. Ultrasonography (US) is the first-line imaging modality for assessing scrotal status and identifying pathologies because of its ease of use, expediency, and high diagnostic sensitivity and specificity. In this case series, we describe the different sonographic features of acute scrotal pathologies seen in emergency settings.

## Introduction

Ultrasonography (US) is a non-invasive diagnostic tool that allows physicians to visualize anatomic structures within the body. It utilizes transducers and probes that emit ultrasound waves that lie between 2 and 18 Megahertz, a range above the human hearing level [1]. The transducers are composed of piezoelectric materials made from ceramic crystals. Through this, a beam of waves is transmitted, and waves are reflected by materials they encounter, such as internal organs, bone, and tissue. There are variations in US, including Doppler, grayscale, and contrast-enhanced, that allow for improvements in resolution according to the needs of the case [2].

US is non-invasive and easily used to evaluate air or fluid-filled structures, especially in emergency settings. Though the clarity of imaging results is highly dependent upon the experience and training of the ultrasonographer, it is highly cost-effective and may be considered the first-line diagnostic option for scrotal emergencies, such as testicular torsion, epididymitis, traumatic injuries, and infectious pathologies, such as Fournier gangrene [3].

The scrotum is a thin sac composed of smooth muscle and skin. It is divided into two segments and encapsulates the testes, epididymis, and the spermatic cord. It plays a significant role in the thermoregulation of the testes, which is crucial for spermatogenesis [4].

Vascular

Testicular Torsion

Testicular torsion can present in two forms: extravaginal and intravaginal. Extravaginal occurs outside the tunica vaginalis and is presently most commonly seen in newborn males [5]. Intravaginal torsion occurs most commonly in adolescent or adult males, within the tunica vaginalis [5]. Long and narrow mesentery or a bell-clapper deformity may predispose men to intravaginal torsion [5]. Patients often present with acute pain accompanied by nausea, vomiting, low-grade fever, and an absent cremasteric reflex. It occurs in one of 4,000 men younger than the age of 25. The sensitivity of US to detect testicular torsion is 0.86. US, in relation to ruling out a diagnosis of testicular torsion, has a specificity of 0.96 [5]. The most common symptoms are unilateral, moderate, or severe scrotal pain. Many patients also report severe abdominal pain. Torsion can be complete, incomplete, or transient [6]. Sonography is the best imaging tool for diagnosis. However, in Doppler imaging and grayscale, epididymo-orchitis and testicular detorsion syndrome may appear similar [6,7].

Testicular Appendage Torsion

Examination findings in patient cases suspicious for testicular appendage torsion include acute scrotal pain with a preserved cremasteric reflex [8]. The symptoms and presentation are very similar to testicular torsion. However, the tenderness is located more superiorly on the testicle in cases of testicular appendage torsion. A palpable, small, and firm nodule with blue discoloration, known as the "blue dot" sign, is found on physical examination [8]. Most cases of testicular appendage torsion, ranging from 91% to 95%, involve the appendix testis [9].

Thrombosed Varicocele

Varicoceles are found in 10-15% of healthy adult males, with higher percentages in infertile males. They commonly occur in teenagers and young adults and are rarer in the older male population. Incidence of thrombosed varicocele increases by 10-20% in individuals who are between the ages of 10 and 19. This can be due to increased body mass and overall increased blood flow to the testicles. Pathophysiological causes of varicoceles include high pressure on the veins and incompetence of the venous valve system, which can cause a reflux that can result in venous dilation [10,11]. Left-sided varicoceles are more common than right-sided varicoceles, due to lower blood flow within the left spermatic vein [12]. Thrombosed varicoceles can occur spontaneously or postoperatively and can occur spontaneously because of trauma or coagulation abnormalities [13]. US can be used as an imaging modality, and images can show a hematoma that surrounds the distended left spermatic veins [14].

Testicular Ischemia Secondary to Extrinsic Compression 

Testicular ischemia can be caused by compression against a hydrocele. This is a rare pathology and has been reported only occasionally in cases of testicular trauma. Large and tense hydroceles require early surgical repair, as they are highly unlikely to self-resolve spontaneously with increased hydrocele size.

Traumatic

Traumatic Hematomas

Hematomas may occur intratesticularly or extratesticularly. After blunt scrotal injury, extratesticular hematomas are a common finding, and the third most common cause of scrotal pain [15]. Imaging presentations demonstrate blood collections inside the tunica vaginalis and testicular hematoceles that, on physical exam, present as painful hard masses [15].

Testicular Rupture

Testicular rupture is common between the ages of 15-40 years, often caused by blunt injury through scenarios such as motor vehicle accidents, assaults, and athletic injuries. The normally echogenic tunica albuginea is disrupted and necessitates emergent surgical intervention [16]. Ultrasonographic diagnosis has a sensitivity and specificity of over 95%. Delayed diagnosis of testicular rupture can result in loss of spermatogenesis, ischemia, abscess, necrosis, and abscess formation [16].

Testicular Fracture

Testicular fractures have a low incidence rate but are still considered a scrotal emergency due to the severity of the situation, as they can lead to erectile dysfunction or testicular loss. The clinical presentation is a sudden cracking or popping noise. This is then followed by detumescence, which leads to pain as well as swelling [17]. Another common clinical presentation is a hematoma of the penis. However, if the Buck’s fascia is torn, there may be excessive swelling leading to a butterfly or eggplant sign in the perineum [18]. Testicular fractures are treated conservatively if US identifies normal blood flow [17,18]. If flow is absent, emergent surgery is required [18].

Pneumoscrotum

Pneumoscrotum is a rare condition usually arising in middle-aged and geriatric males that is critical to diagnose, as it can have significant morbidity and mortality [18]. Very rarely, cases can present in male infants [19]. The term is used to define the presence of air within the scrotum. Clinical manifestations include swelling of the scrotum, palpable crepitus, and urinary retention [20]. 

Infectious

Epididymo-Orchitis

*Chlamydia trachomatis* and *Neisseria gonorrhea *are the most common causes of epididymo-orchitis in adolescents. In prepubertal boys and men above the age of 35, *Escherichia coli *and *Proteus mirabilis* are responsible for the majority of cases. Rarer causes include sarcoidosis, tuberculosis, mumps, and amiodarone hydrochloride [21]. 

Abscesses

Scrotal abscesses are rare and mostly occur in men aged 15-35. Epididymo-orchitis is a precipitating factor development of epididymal abscesses. Immunocompromised individuals are at higher risk and may develop abscesses secondary to tuberculosis or fungal infections [21]. Clinical manifestations include acute scrotal pain as well as swelling or inflammation. US is used for imaging, and the presence of an abscess necessitates surgical intervention [22]. 

Pyocele

Pyocele can present secondarily as a consequence of untreated epididymo-orchitis or rupture of an intratesticular abscess into the space between the layers of the tunica vaginalis [23]. This is most common in older male adults [23]. Clinical manifestations include swelling, pain, and discharge [24,25]. US is the primary imaging method for pyocele [26]. 

Tuberculous Epididymo-Orchitis

Tuberculous epididymo-orchitis is the second most common manifestation of extrapulmonary tuberculosis, with a prevalence of 400 per 100,000 individuals [27]. Clinical manifestations include an enlarged right tactical with intratesticular masses, as well as a swollen spermatic cord. An inflamed epididymitis may also be present [28]. Tuberculosis orchitis typically results from neighboring extension from the epididymis. Presentation is usually unilateral [28].

Fournier Gangrene

Fournier gangrene consists of necrotizing fasciitis of the scrotum, perianal region, and perineum [29]. Causes are polymicrobia. Fournier's gangrene constitutes a true surgical emergency, as the mortality rates range up to 50%. Mortality can reach 75% if the diagnosis is delayed [30]. Clinical presentation is commonly used for diagnosis; however, if there is an ambiguous presentation, US may be required. Subcutaneous gas is a stereotypical finding, but its absence of subcutaneous gas does not exclude Fournier gangrene as a possible diagnosis [29,30].

## Materials and methods

To understand the sonographic features of acute scrotal emergencies, we retrospectively reviewed clinical cases from the image network of University of Florida Health - Jacksonville between 2015 and 2025 using the mPower search engine. Inclusion criteria included male adults ≥18 years, with initial search terms limited to ultrasonographic imaging with “acute” and “emergency” in the impressions sections of results. Exclusion criteria included non-ultrasound imaging, outpatient examinations, and cases without overt presentations.

These search parameters generated over 200 results, which were then filtered according to each pathology. Representative cases were selected from these subsets that were subjectively deemed to best represent the specific diagnoses listed above, as directed by the senior-most attending physician.

Permission to use the selected images was obtained from each patient whose pathology was selected for inclusion in this case series. Data points from each case, including demographics, imaging findings, and results, were obtained and compiled. Images were then deidentified and stored on secure network computers accessed only by study personnel. This case series was then conducted with the notable imaging features described in the reports.

## Results

Images were collected for 13 of the scrotal pathologies that we describe. All patients identified as “white” and assigned male at birth in their self-reported demographics. No patient presented with multiple of the described pathologies (Table 1).

**Table 1:** Patient demographics for each selected case of scrotal trauma.

Case Type	Race	Age	Notable Features
Testicular torsion	White	19	Acute scrotal pain with nausea and vomiting
Testicular appendage torsion	White	22	Acute scrotal pain with preserved cremasteric reflex
Thrombosed varicocele	White	25	Acute scrotal pain with known varicocele
Testicular ischemia secondary to extrinsic compression by hydrocele	White	30	Acute scrotal pain and swelling post-motor vehicle accident
Traumatic hematoma	White	20	Blunt traumatic injury during soccer game
Testicular rupture	White	18	Blunt injury while riding a bicycle
Testicular fracture	White	23	Gunshot wounds to pelvis and scrotum
Pneumoscrotum	White	41	Acute scrotal and lower limb injury post-fall
Epididymo-orchitis	White	19	Retroperitoneal abscess post colonic perforation
Abscess	White	25	Untreated longstanding sexually transmitted infection (STI)
Pyocele	White	60	Untreated urinary tract infection (UTI)
Tuberculous epididymo-orchitis	White	74	Untreated Tuberculosis (TB) infection in recent immigrant
Fournier gangrene	White	51	Immunocompromised patient with metastatic rectal cancer

Vascular

Testicular Torsion

**Figure 1:**
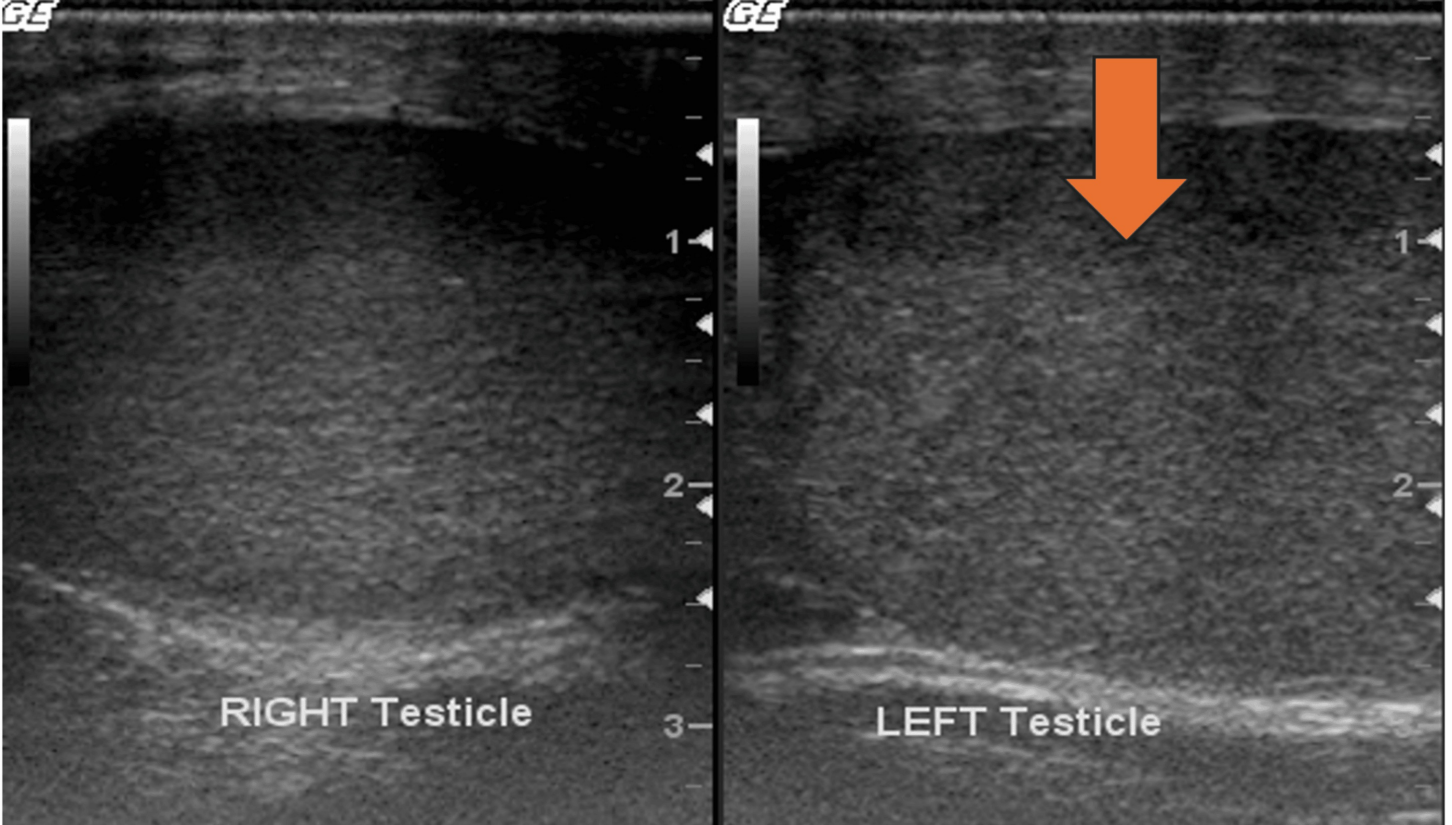
Grayscale US images of the testes illustrates left testicular enlargement (red arrow).

**Figure 2:**
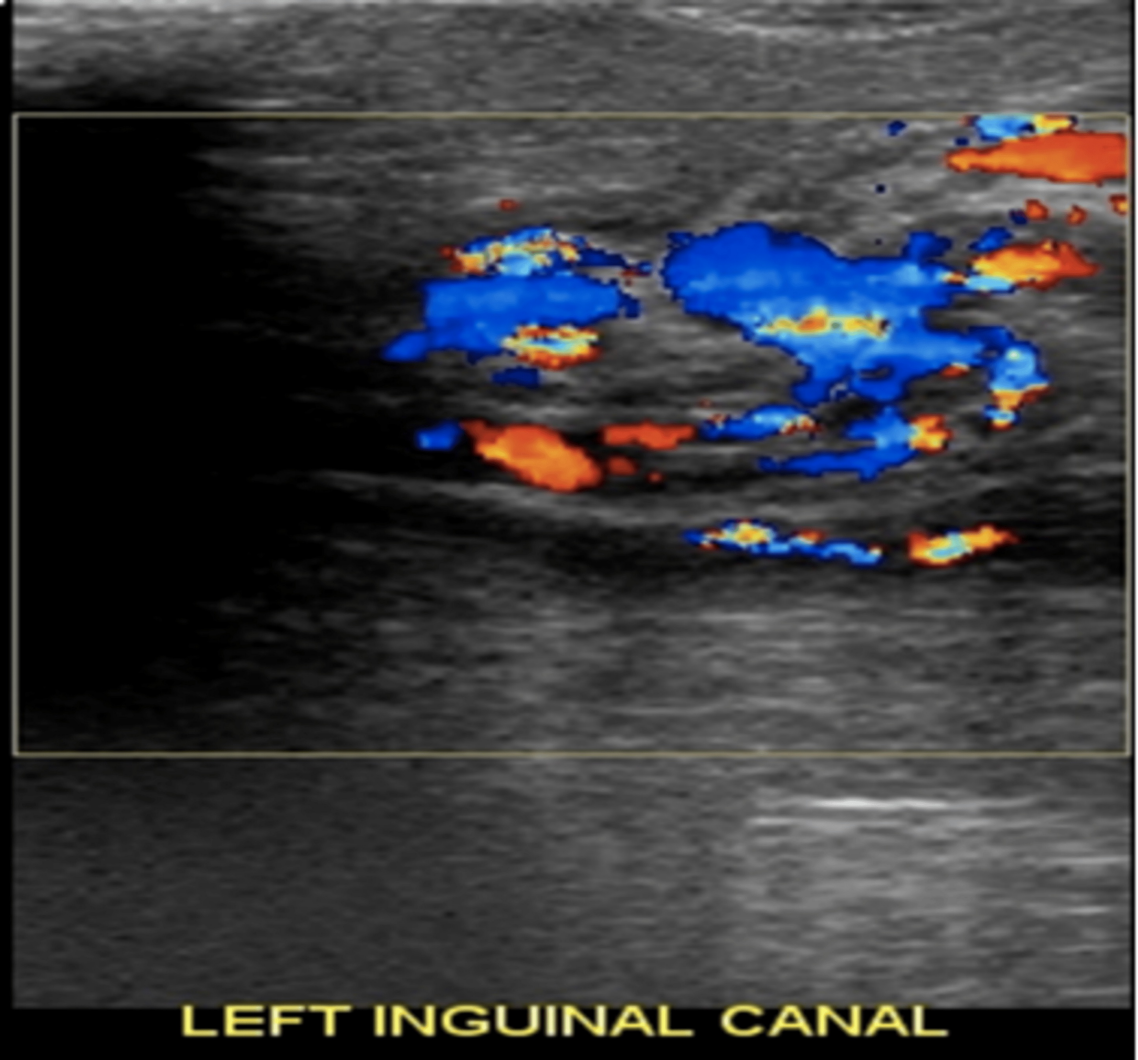
"Torsion knot sign" on color Doppler US of torsed vessels in the inguinal canal.

**Figure 3:**
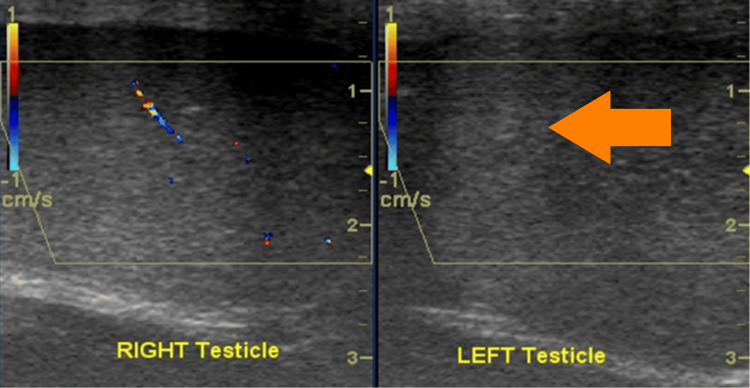
Loss of flow within the left testicle shown on color Doppler US (orange arrow).

**Figure 4:**
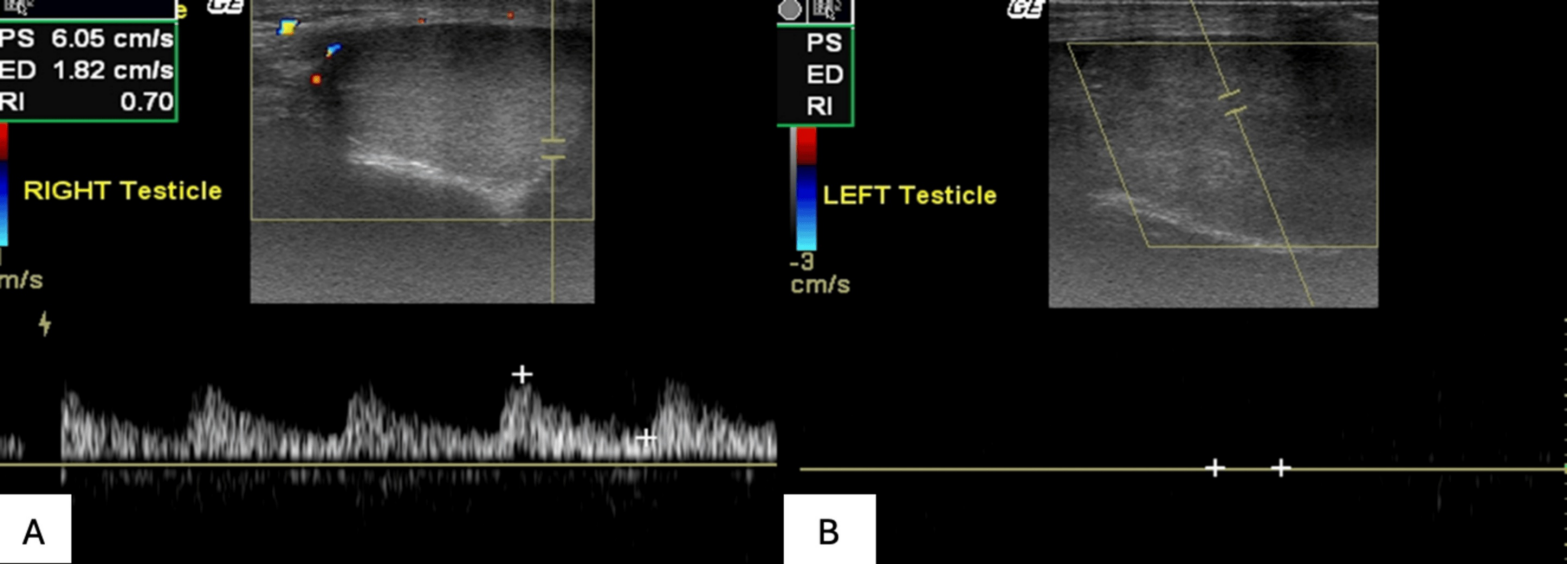
Normal right testicular waveform (4A) and the lack of left testicular waveform (4B) illustrated on power Doppler US.

Gray-scale images (Figure 1) of testicular torsion may appear nonpathologic if the torsion is recent. Within four to six hours, testicular swelling and decreased flow are present. The testis will have a heterogeneous echotexture at 24 hours post torsion. Within the external inguinal ring, there is sometimes a twisted spermatic cord (torsion knot sign), which can cause the spermatic cord to change shape and present as an oval mass. Absent testicular flow on US is a hallmark of ischemia (Figures 2-4). 

Testicular Appendage Torsion

**Figure 5:**
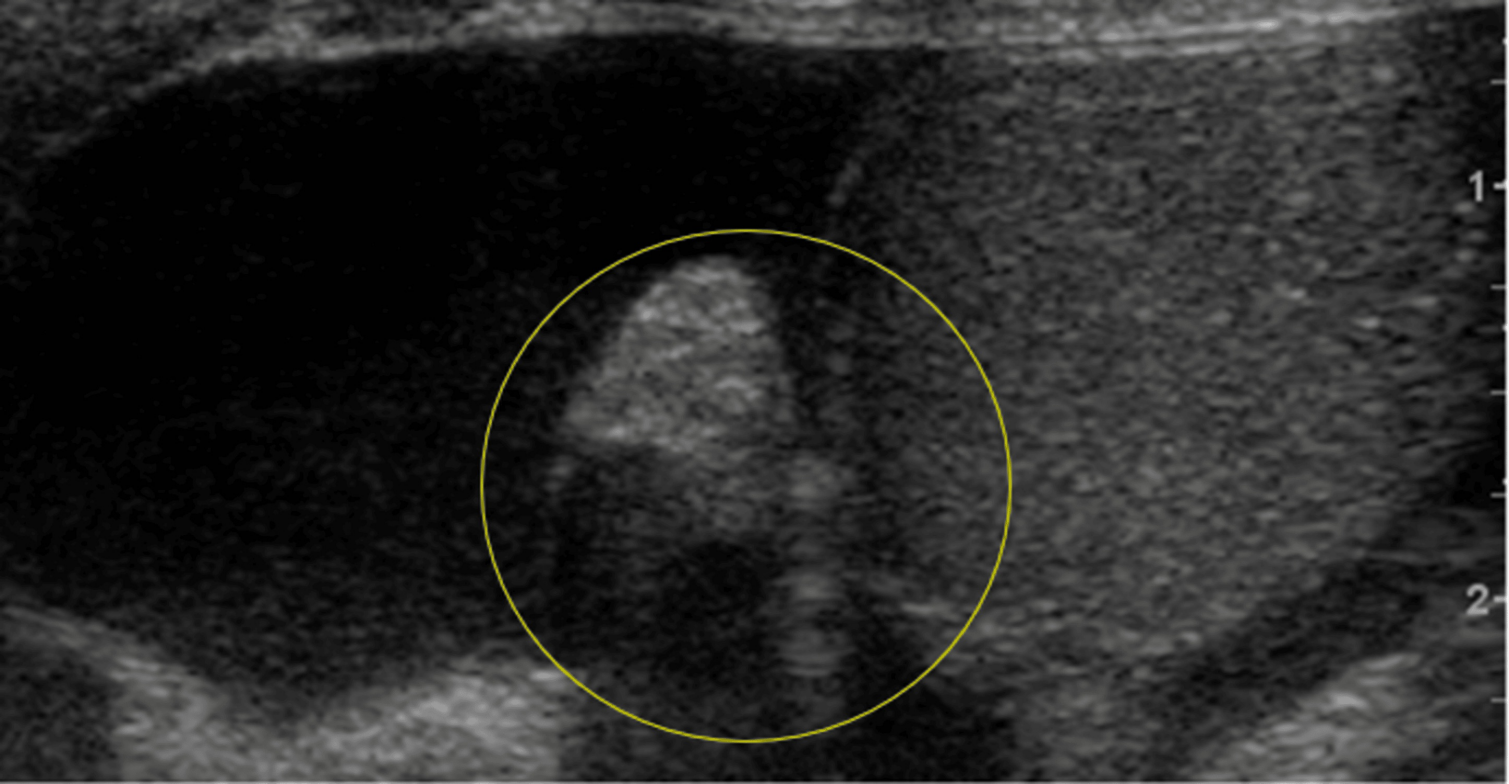
Increased echogenicity and enlargement of the left testicular appendage with a juxtaposing hydrocele seen on grayscale US (yellow circle).

**Figure 6:**
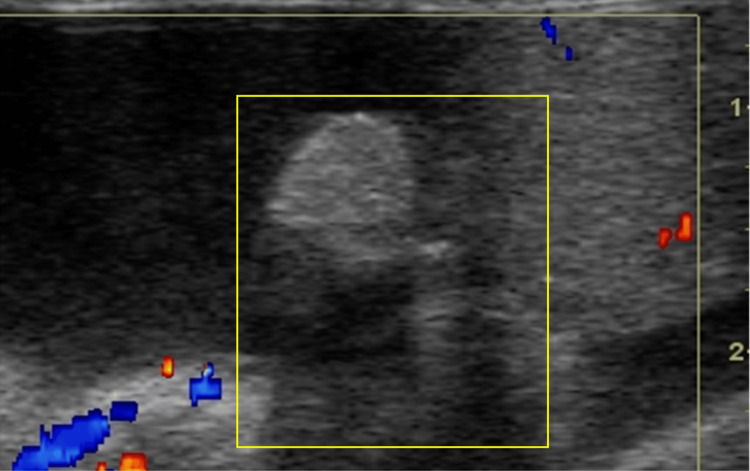
Color Doppler US illustrates the absence of vascularity within the appendage (yellow square).

Ultrasound imaging demonstrates a hyperechoic mass with a nuclear hypoechoic space (Figure 5) next to the testis or epididymis in cases of suspected testicular appendage torsion. Color Doppler US may demonstrate increased blood flow around the contorted testicular appendage (Figure 6). 

Thrombosed Varicocele

**Figure 7:**
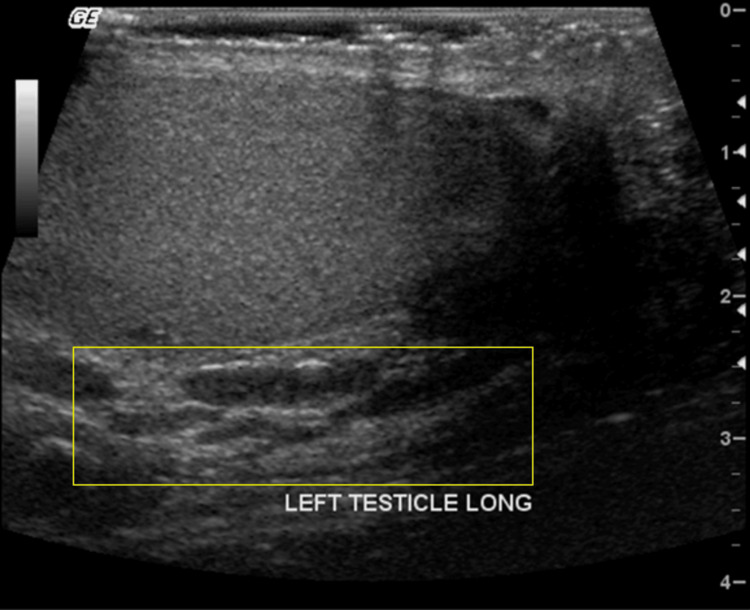
Grayscale US demonstrates a poorly echogenic cordlike dilatation (yellow rectangle) of the pampiniform plexus.

**Figure 8:**
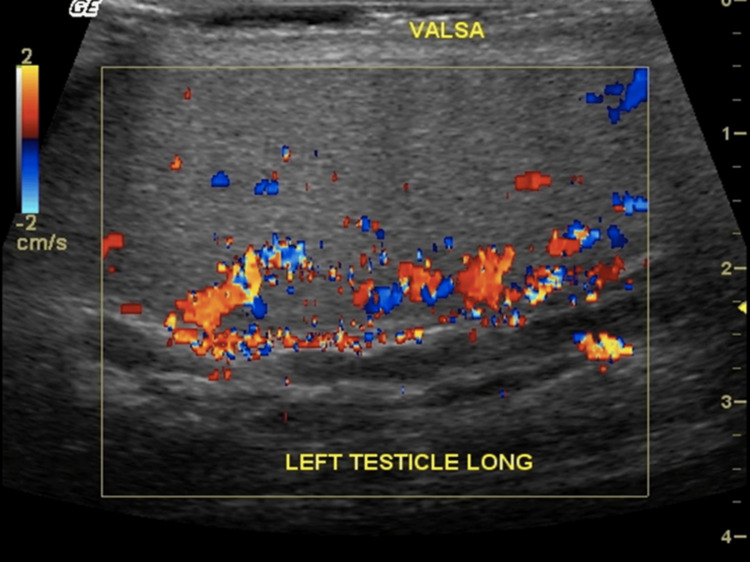
Absent vascular flow within a thrombosed varicocele shown on color Doppler US.

Varicoceles generally do not present as acutely painful. Sometimes, a vein within the pampiniform plexus or the cremasteric plexus can acutely and painfully thrombose (Figures 7-8).

Testicular Ischemia Secondary to Extrinsic Compression

**Figure 9:**
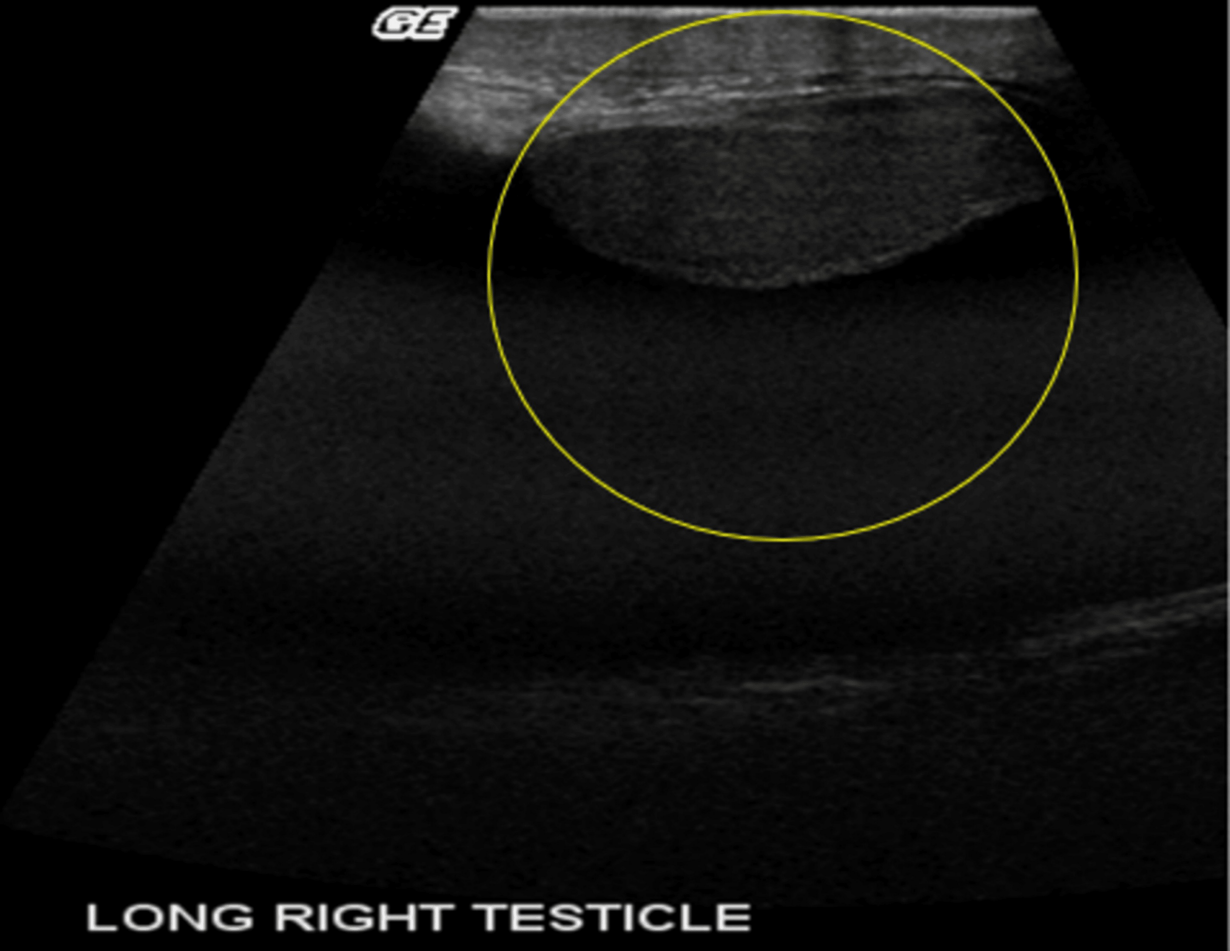
Grayscale US demonstrates large simple appearing hydrocele compressing and marginalizing the testicle (yellow circle).

**Figure 10:**
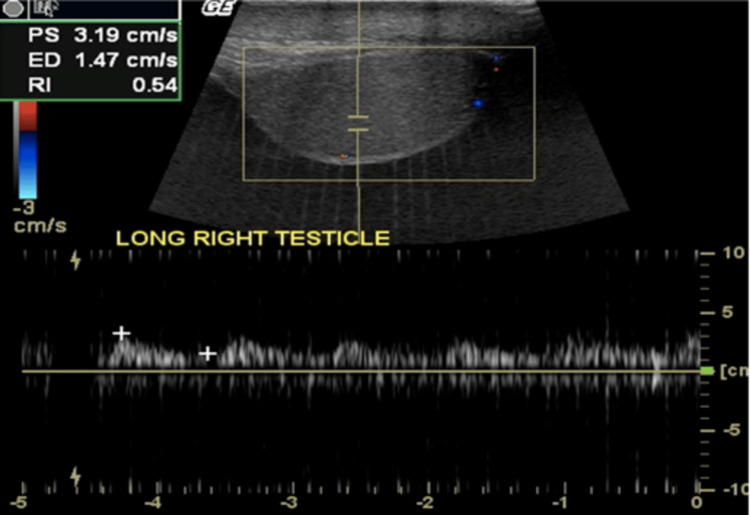
Color US demonstrates a muted systolic waveform with low amplitude diastolic flow and lack of vascularity, which signal acute ischemia (yellow rectangle).

A heterogeneous and asymmetrical appearance of the compromised testicle with reduced diastolic flow reversal can be seen on grayscale US (Figures 9-10).

Traumatic

Traumatic Hematomas

**Figure 11:**
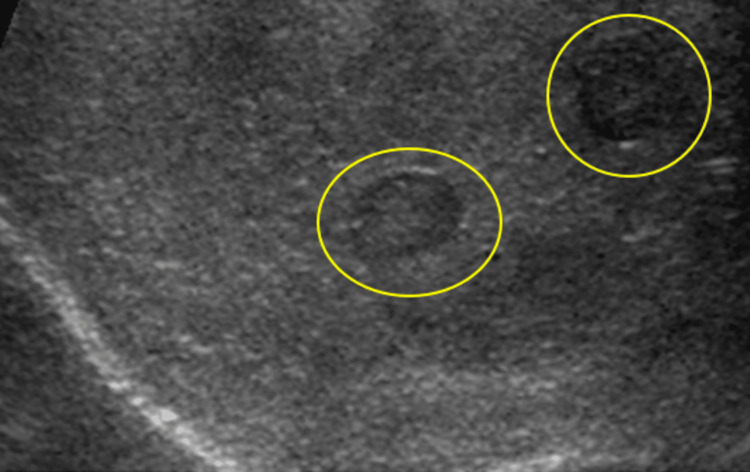
Hypoechoic testicular lesions (yellow circles) with hyperemia of the testicular tissue on grayscale scrotal US.

**Figure 12:**
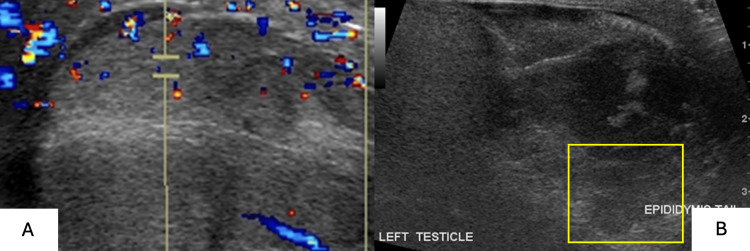
Color (12A) and grayscale (12B) scrotal ultrasonography illustrates a heteroechoic and poorly defined, avascular lesion within the epididymal tail (yellow rectangle).

**Figure 13:**
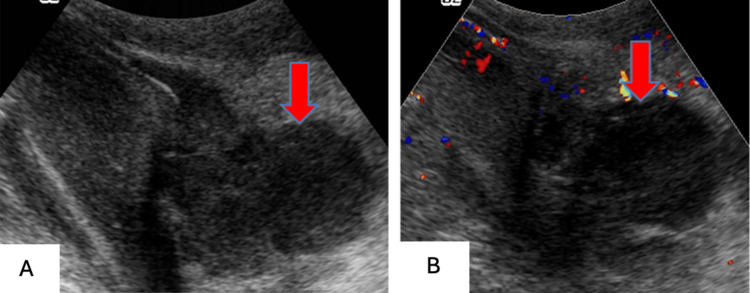
An anechoic and avascular pooling within the scrotal wall shown on grayscale (13A) and color Doppler (13B) US images (red arrows).

Male patient age can cause a varied appearance of acute traumatic testicular hematomas, which may present as isoechoic or hyperechoic and internally avascular compared to testicular tissue (Figure 11). If a hematoma is suspected clinically, repeat imaging within 24 hours to evaluate possible echogenic changes is required (Figures 12A-12B). Chronic hematomas appear anechoic or hypoechoic compared to surrounding testicular tissue (Figures 13A-13B). Color Doppler US will show avascularity, a hallmark feature that differentiates hematomas from tumors; however, peripheral vascularity can develop if subsequent infection occurs.

Testicular Rupture

**Figure 14:**
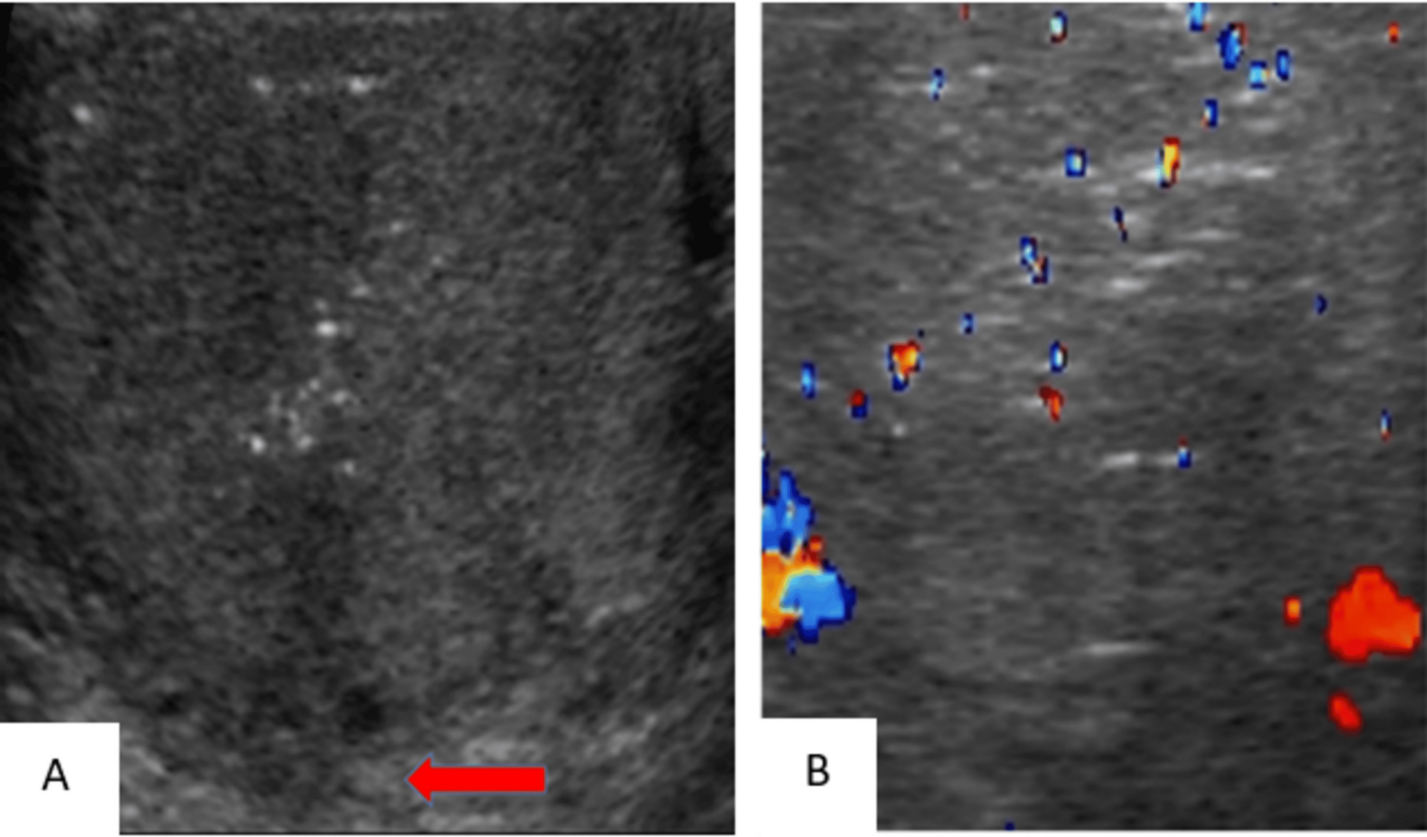
Testicular rupture with broken continuity of the tunica albuginea and irregularly outlined lower pole as seen on grayscale US (14A). Absent blood flow within a hematoma juxtaposed with preserved blood flow to the surroudning testicular tissue shown on color Doppler US (14B).

Patients experiencing testicular rupture can present with profuse swelling and bleeding, with hemorrhagic ejection into the scrotal sac of testicular components. Color and duplex Doppler US can show varied blood flow to the involved testicle (Figures 14A-14B). 

Testicular Fracture

**Figure 15:**
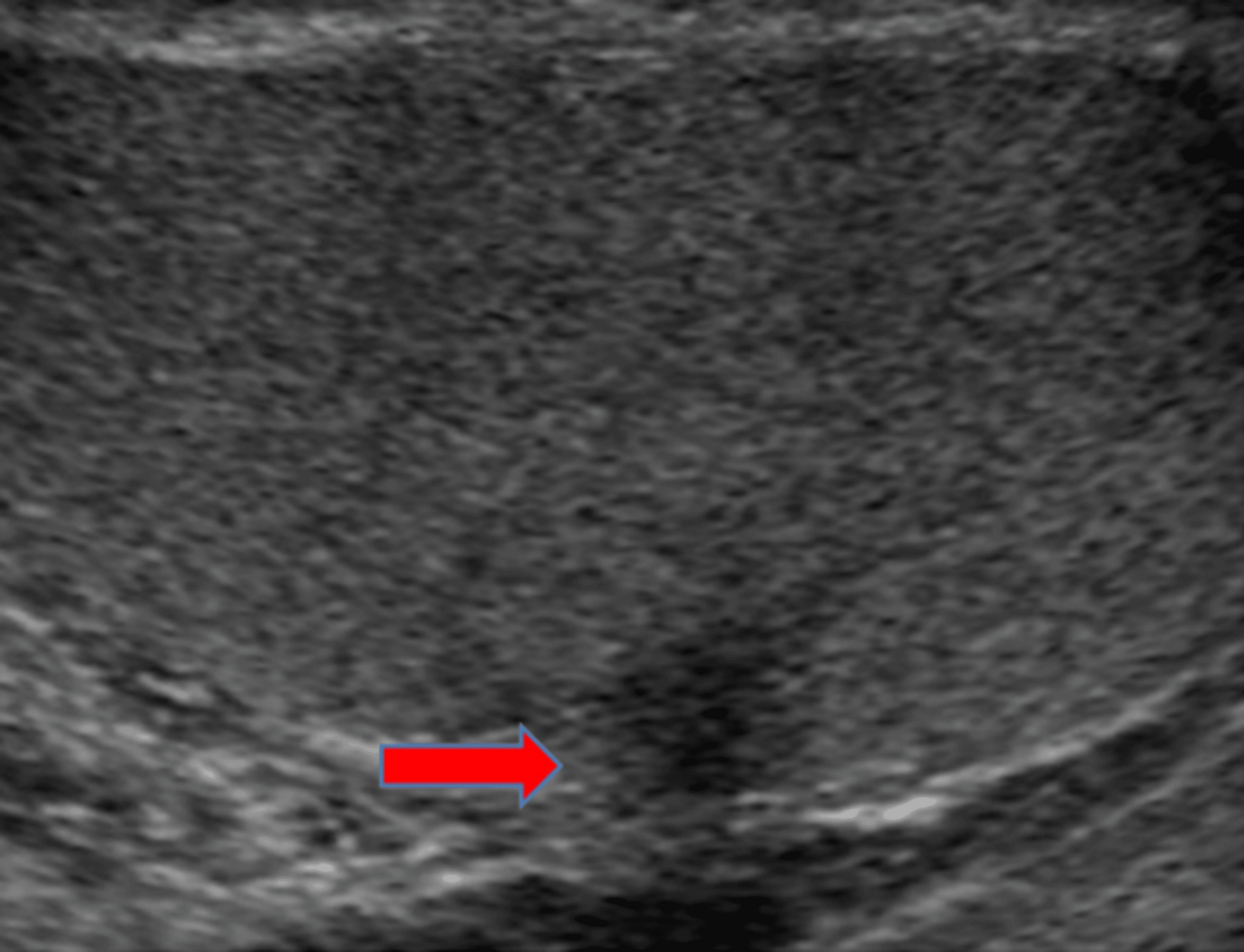
Grayscale image of the left testicle demonstrates an irregular linear hypoechoic band at the inferior aspect of the testicle (red arrow).

**Figure 16:**
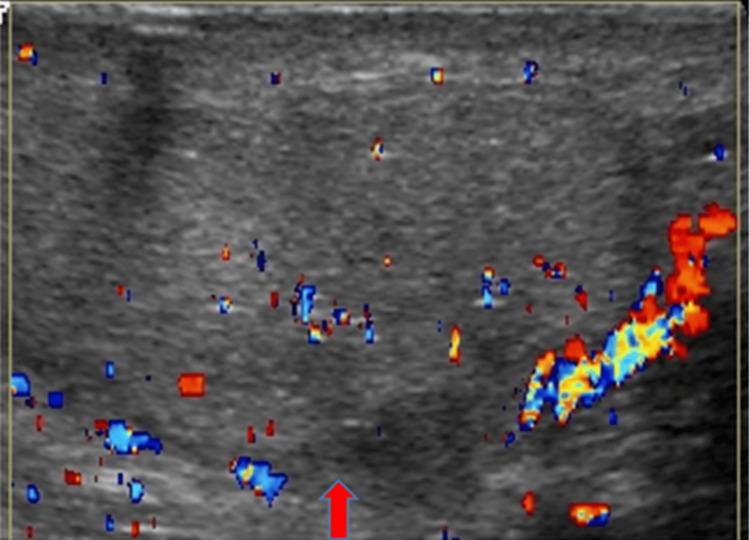
Color Doppler image shows normal flow throughout the testis, except in the area of the fracture (red arrow).

US imaging of a testicular fracture can show a linear hypoechoic band within the affected testicular parenchyma, which represents a break in the normal testicular architecture (Figure 15). Doppler US imaging is used to assess vascular integrity (Figure 16). 

Pneumoscrotum

**Figure 17:**
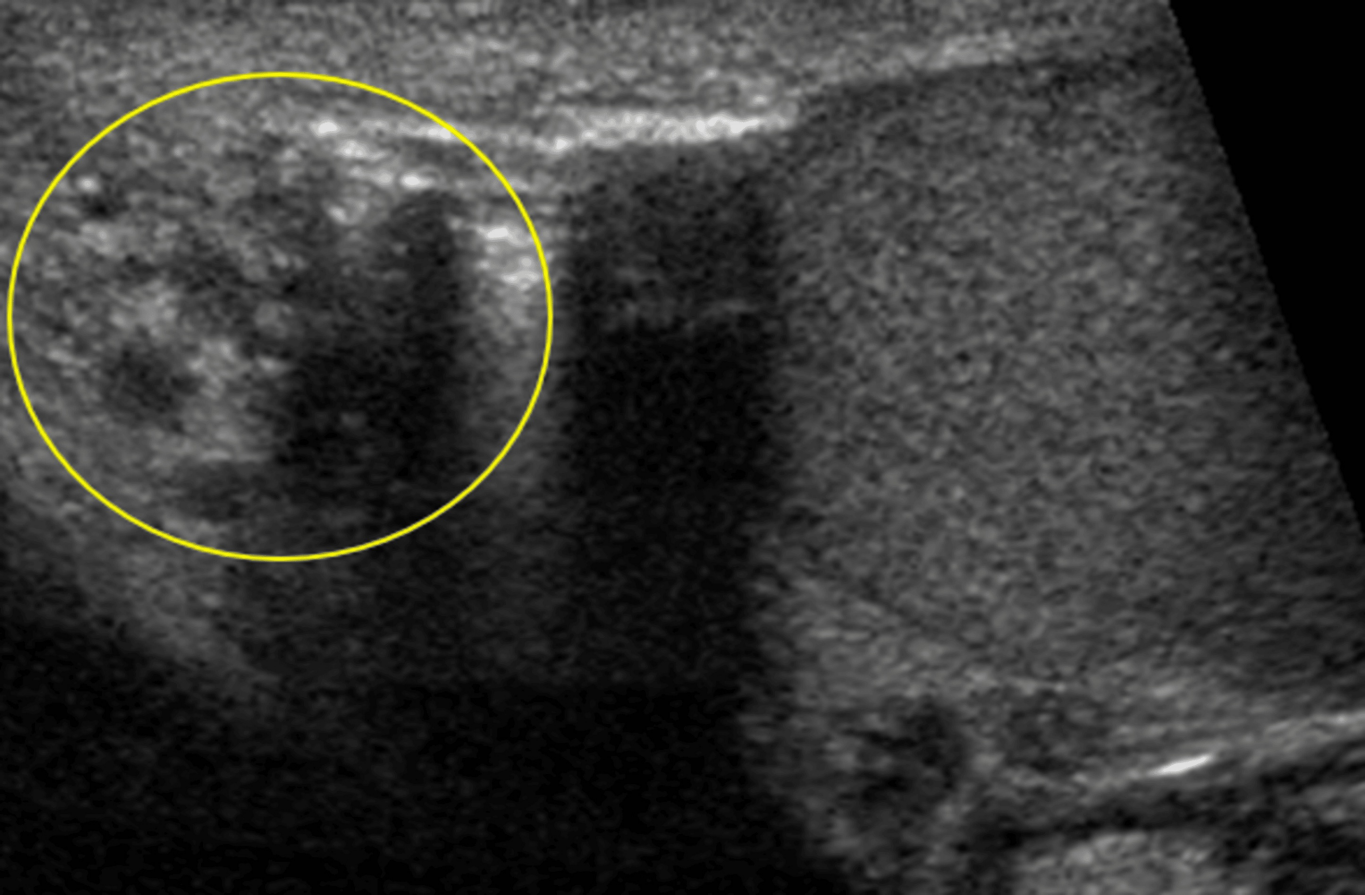
Grayscale image demonstrating large gas collection within the scrotal sac seen as hyperechoic foci with dirty shadowing (yellow circle).

Ultrasonography of a pneumoscrotum patient will demonstrate air arising from trauma or gas-producing infectious organisms (Figure 17).

Infectious

Epididymo-Orchitis

**Figure 18:**
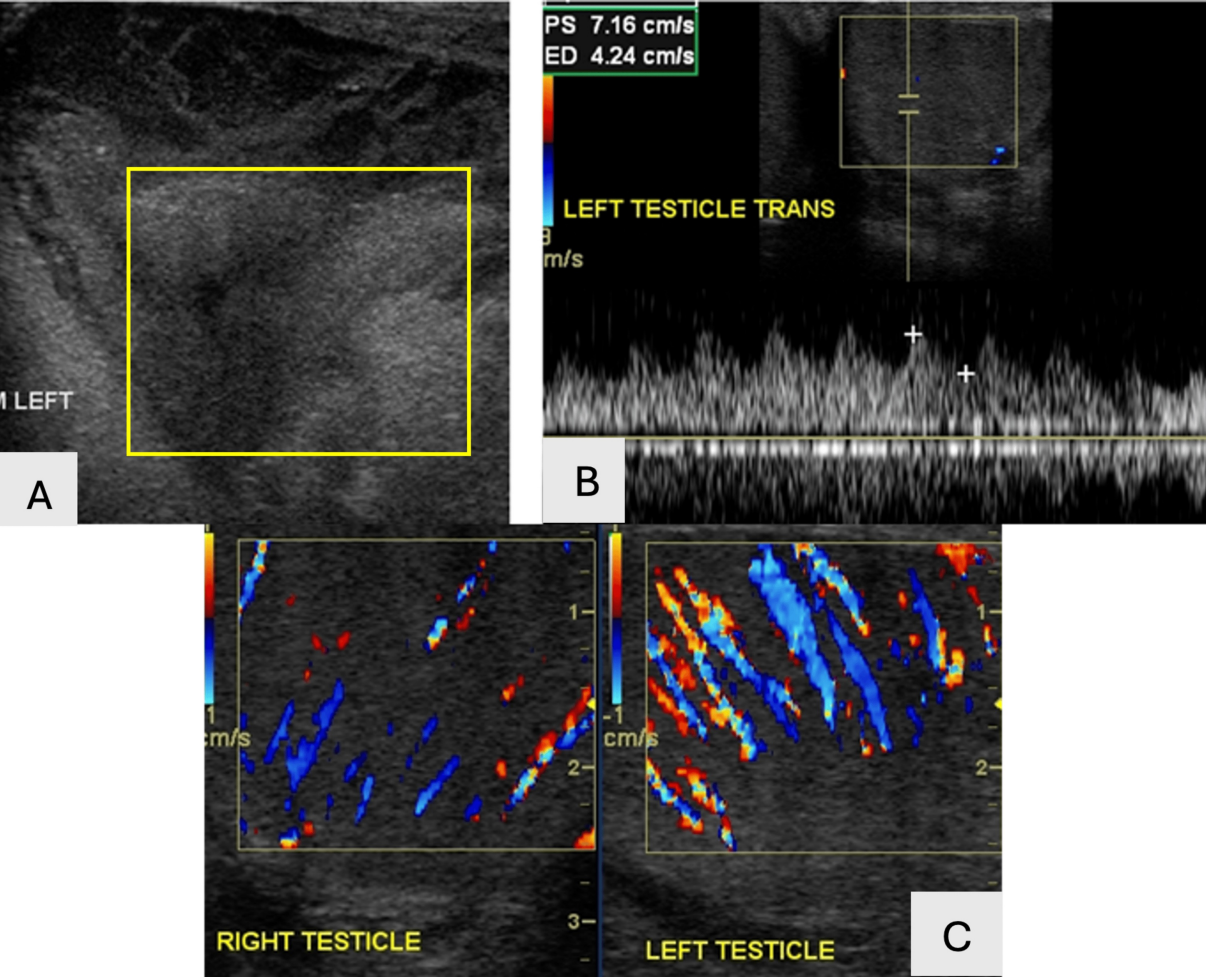
Grayscale (18A) (yellow rectangle) and color Doppler (18B and 18C) US images demonstrate left epididymal head enlargement with decreased vascular resistance and increased diastolic flow on spectral waveform.

A hyper- or hypoechoic and enlarged epididymis, secondary to hemorrhage, will be visible on grayscale US (Figures 18A-18C). Hyperemia of the epididymis and testis is present on color Doppler US. Decreased vascular resistance and increased diastolic flow are seen on the spectral waveform.

Abscesses

**Figure 19:**
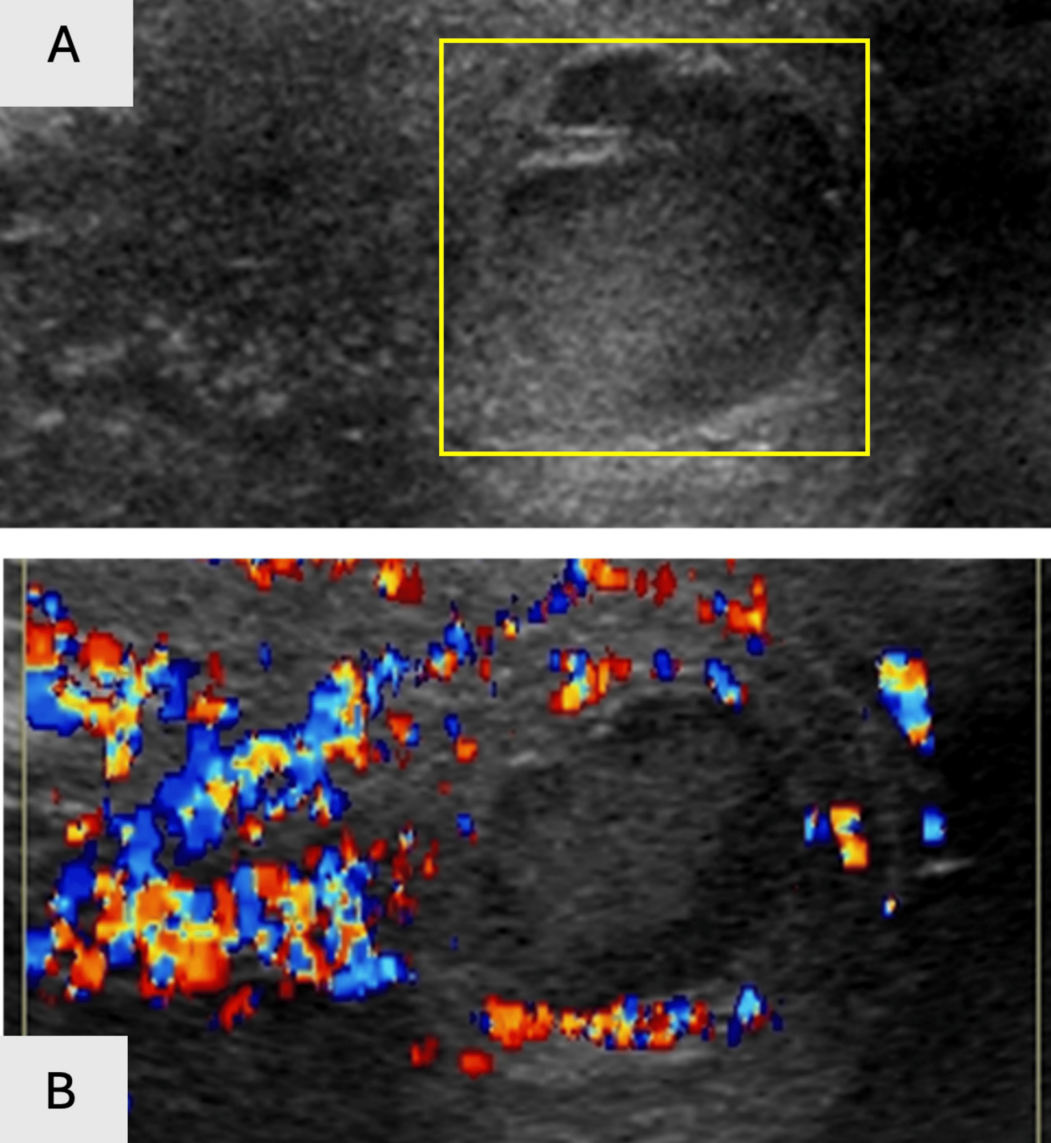
Gray scale (19A) (yellow rectangle) and color Dopper (19B) US demonstrate increased epididymal vascularity, with a slightly echogenic lesion with the outer vascularity.

**Figure 20:**
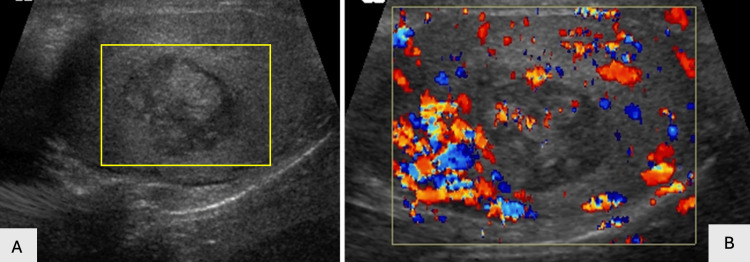
Testicle with a central echogenic abscess (yellow rectangle) on grayscale US imaging (20A). Increased vascular flow surrounding the central testicular abscess seen on color Doppler US (20B).

Abscesses present with irregular walls and minor internal echoes. Peripheral vascularity is also sometimes present (Figures 19A-19B and Figures 20A-20B). 

Pyocele

**Figure 21:**
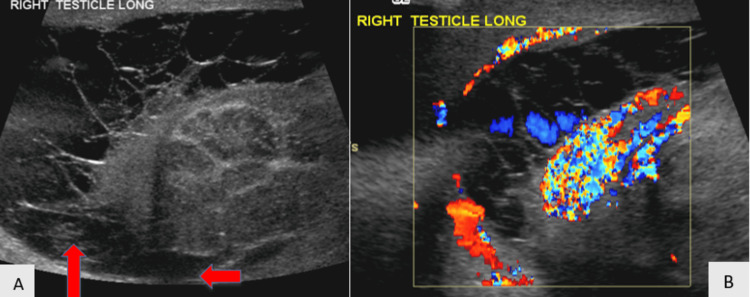
Pyocele (red arrow pointing upward) and abundant intratesticular abscesses with increased scrotal wall blood flow (red arrow pointing left) seen on grayscale US imaging (21A). Complicated pyocele illustrated on color Doppler US (21B).

US imaging findings in suspected pyocele include a "falling snow sign," loculations, septations, or the presence of gas (Figures 21A-21B).

Tuberculous Epididymo-Orchitis

**Figure 22:**
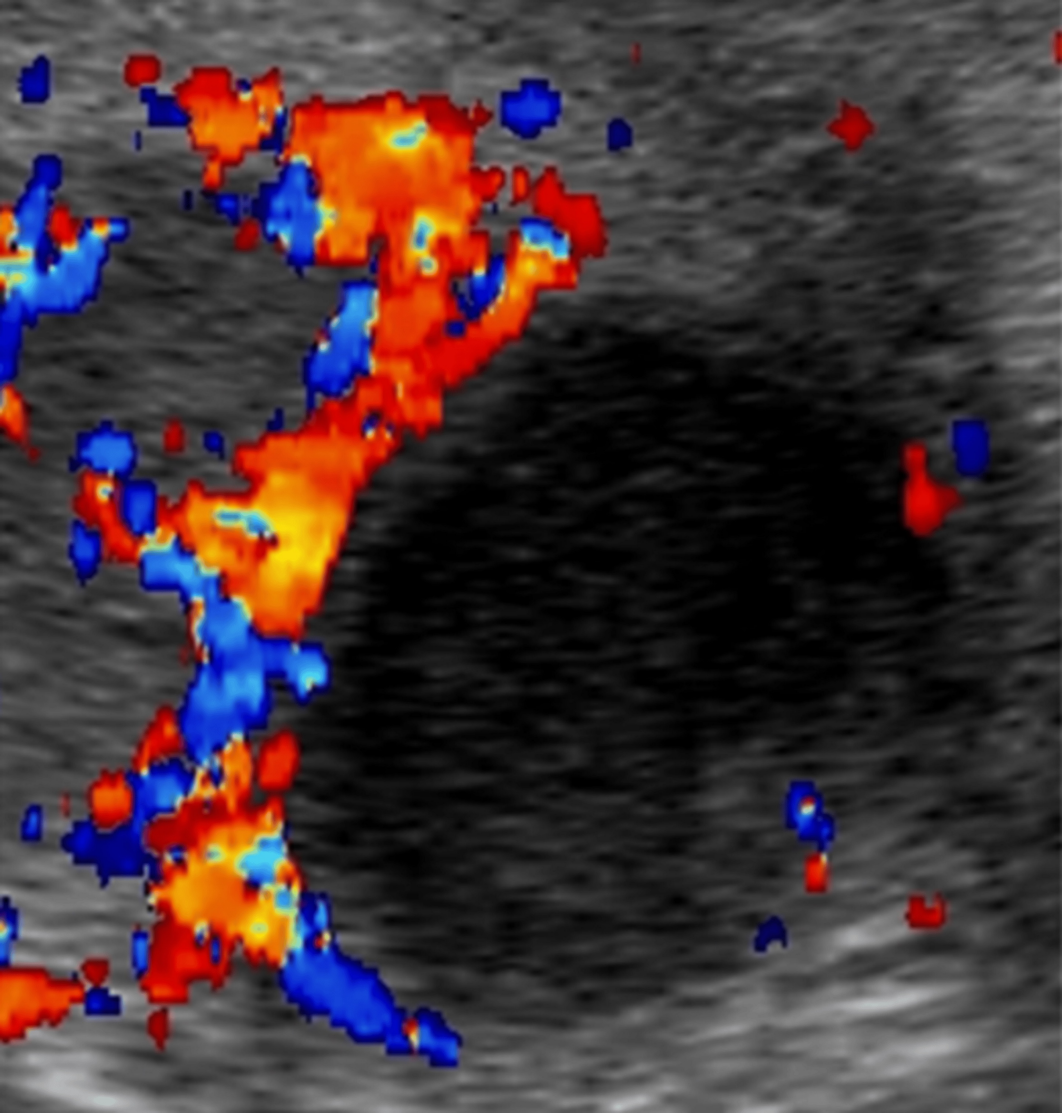
Testicular necrosis secondary to active TB infection, with increased surrounding vascular flow, as shown on Color Doppler US.

**Figure 23:**
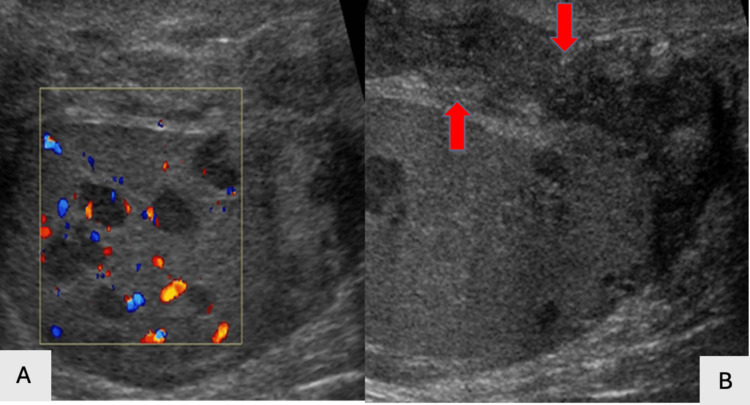
Color Doppler (23A) and grayscale (23B) images through the testicle demonstrate multiple microabscesses in a TB patient (yellow rectangle and red arrows).

Tuberculous epididymitis presents on US imaging with generalized epididymal enlargement and decreased echogenicity (Figure 22 and Figures 23A-23B).

Fournier Gangrene

**Figure 24:**
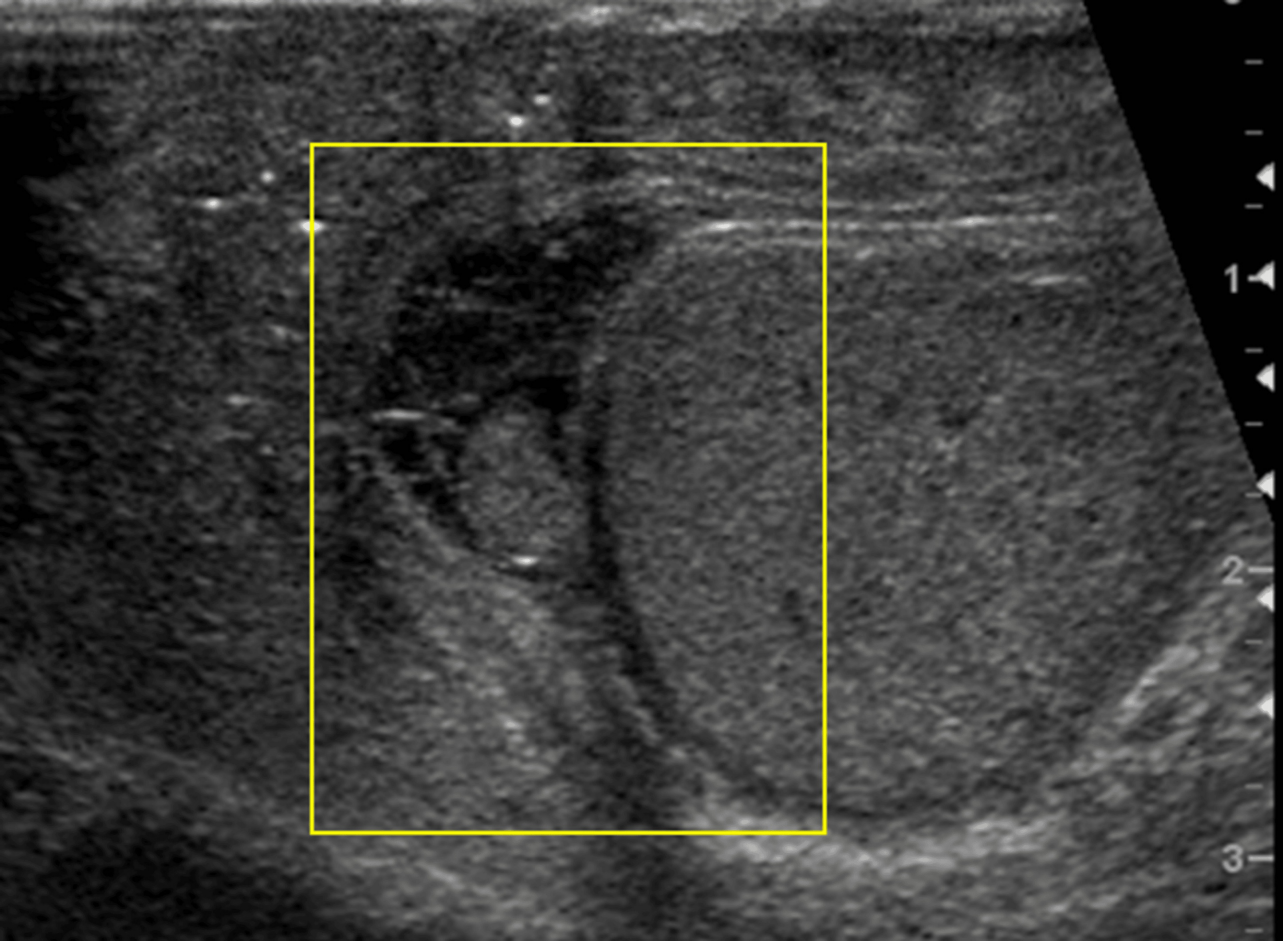
Gaseous collections and increased scrotal wall size (yellow rectangle) on grayscale US imaging of a patient with Fournier gangrene.

Sonographic findings of Fournier gangrene include scrotal wall thickening with subcutaneous gas present (Figure 24).

## Discussion

US is imperative for the efficient and thorough diagnosis of acute scrotal emergencies. This review reinforces prevalent medical approaches to suspected testicular crises. The described imaging features for the most common and potentially most dangerous scrotal emergencies align with reported findings in previous reviews. However, this review is more comprehensive in its approach to testicular emergencies and includes pathologies that are often not discussed because they are less prevalent in Western society, such as tuberculous epididymo-orchitis. While such pathologies might not be as prevalent within certain populations, because of their potential morbidity and mortality related to delayed diagnosis, it is still imperative that the clinical radiologist be able to recognize them expediently.

While the described pathologies may all benefit diagnostically from the use of ultrasonography, in several cases, US is not the recommended initial imaging modality. If there is a high index of clinical suspicion for the presence of gas formation within the scrotum, computed tomographic (CT) imaging or X-ray imaging is recommended to diagnose pneuomoscrotum [18,19]. CT imaging is also the most specific modality for diagnosing Fournier gangrene. US is useful in ruling out the presence of air within the scrotum. However, US should be followed by confirmatory CT imaging [29,30].

In many instances, certain features present on US warrant surgical correction, regardless of additional imaging to confirm a specific pathology. For example, the presence of air within the scrotum on US will always require surgical intervention, even if Fournier gangrene, pneumoscrotum, or abscess has not been exclusively diagnosed [30].

Limitations

A key limitation of this case-based review is potential selection bias due to the retrospective nature of the case and image collection process. In addition, variability in ultrasound technique can impact image acquisition and diagnostic accuracy for acute scrotal pathologies. Furthermore, this review presents a select number of cases rather than a comprehensive cohort and may not include the full spectrum of variability seen in some acute scrotal pathologies. Finally, because the images have been gathered from one institution, these may not represent the full diversity of presentations found in broader clinical practice. Therefore, while this review provides valuable information regarding ultrasonographic evaluation of acute scrotal emergencies, it should be interpreted within the context of these limitations.

## Conclusions

Given the expeditious diagnostic utility of US, it is commonly the first-line imaging modality for scrotal examination. Many testicular pathologies are easily recognizable based on clinical presentation alone. However, US imaging can help describe the extent and severity of pathologies, especially those secondary to traumatic injury. Because of potentially time-sensitive complications of testicular pathologies, which might permanently impact appearance and function, it is clinically imperative for radiologists to recognize their appearances on US.
